# Serine Hydroxymethyltransferase 2 Deficiency in the Hematopoietic System Disrupts Erythropoiesis and Induces Anemia in Murine Models

**DOI:** 10.3390/ijms252011072

**Published:** 2024-10-15

**Authors:** Jisheng Li, Bowen Zhang, Yunqiao Li, Chuanli Liu, Xuan Tang, Jiahui Zhao, Xuetao Pei, Yanhua Li

**Affiliations:** Beijing Institute of Radiation Medicine, Beijing 100850, China; 13516279671@163.com (J.L.); bowen_0901@126.com (B.Z.); yunqiaoli91@126.com (Y.L.); chuanlili1@163.com (C.L.); tx99524@163.com (X.T.); jiahuizhao2024@126.com (J.Z.)

**Keywords:** serine hydroxymethyltransferase 2, red blood cell, erythroid differentiation, anemia, bone marrow, *Vav1-Cre*

## Abstract

Serine and folate metabolism play critical roles in erythroid development in both embryonic and adult mice; however, the precise roles of these metabolic pathways in erythropoiesis and the pathophysiology of anemia remain inadequately characterized in the literature. To delineate the contributions of serine and folate metabolism to erythroid differentiation, we focused on serine hydroxymethyltransferase 2 (SHMT2), a key regulatory enzyme within these metabolic pathways. Using gene-editing techniques, we created fetal and adult mouse models with targeted deletion of *Shmt2* in the hematopoietic system. Our findings demonstrated that the deletion of *Shmt2* within the hematopoietic system led to the distinctive anemia phenotype in both fetal and adult mice. Detailed progression analysis of anemia revealed that *Shmt2* deletion exerts stage-specific effects on the development and maturation of erythroid cells. Specifically, *Shmt2* deficiency promoted erythroid differentiation in the R2 (CD71^+^ Ter119^−^) cell population residing in the bone marrow while concurrently inhibiting the proliferation and erythroid differentiation of the R3 (CD71^+^ Ter119^+^) cell population. This disruption resulted in developmental arrest at the R3 stage, significantly contributing to the anemia phenotype observed in the models. This study elucidates the critical role of *Shmt2* in erythroid development within the hematopoietic system, highlighting the underlying mechanisms of erythroid developmental arrest associated with *Shmt2* loss.

## 1. Introduction

Anemia is a heterogeneous disorder characterized by diverse types and underlying causes. It can be classified into three primary categories based on red blood cell size: microcytic, macrocytic, and normocytic anemia. Normocytic anemia includes conditions such as osteomyelic anemia, aplastic anemia, anemia associated with chronic diseases, and nephrotic syndrome. Microcytic anemia encompasses sideroblastic anemia, iron deficiency anemia, and thalassemia, while macrocytic anemia is divided into megaloblastic and non-megaloblastic varieties [1]. Megaloblastic anemia is mainly linked to defective DNA synthesis in hematopoietic progenitor cells, leading to ineffective erythropoiesis and bone marrow hemolysis [2,3]. The main causes are deficiencies in folate or vitamin B12 (cobalamin). These vitamins are crucial for one-carbon metabolism, which is necessary for converting deoxyuridine monophosphate (dUMP) to deoxythymidine monophosphate (dTMP). Within the pyrimidine biosynthetic pathway, 5,10-methenyltetrahydrofolate acts as a methyl donor and is then converted to dihydrofolate. This compound requires reduction and remethylation for reutilization [4]. Notably, the conversion of dihydrofolate to tetrahydrofolate is a target for various pharmacological agents, particularly chemotherapeutics like methotrexate, which can cause anemia by inhibiting pyrimidine synthesis [5,6].

Serine hydroxymethyltransferase (SHMT) is critical for synthesizing 5,10-methenyltetrahydrofolate, with serine hydroxymethyltransferase 2 (SHMT2) serving as a pivotal enzyme in mitochondrial serine catabolism. SHMT2 mainly boosts intracellular 5,10-methenyltetrahydrofolate levels by transferring the hydroxymethyl group from serine to tetrahydrofolate, providing the methyl groups needed for Nicotinamide adenine dinucleotide phosphate (NADPH), S-adenosylmethionine (SAM), and pyrimidine synthesis. [7,8]. Given this role, we hypothesize that SHMT2 is crucial for erythroid differentiation and anemia pathogenesis, although few studies have addressed this.

The *Vav Guanine Nucleotide Exchange Factor 1 (Vav1)* gene is expressed throughout all stages of hematopoietic cell development, encompassing myeloid, erythroid, B, and T lymphocytes [9,10,11]. In this study, to explore the hematopoietic system abnormalities and anemia phenotypes arising from the conditional knockout of *Shmt2*, we utilized the Cre-lox recombination system to establish the *Vav1-Cre/Shmt2^fl/fl^* embryonic and adult mouse model. The model was generated by crossing *Vav1-Cre* mice with *Shmt2-flox* mice [9,10,11,12]. Additionally, we aimed to clarify the role and molecular mechanisms of SHMT2 in erythroid cell maturation. This research offers valuable insights into anemia model development and significantly advances our understanding of the disease’s pathophysiology and potential treatments.

## 2. Results

### 2.1. The Absence of the Shmt2 Gene within the Hematopoietic System Significantly Impairs the Development of Erythroid Cells in the Fetal Livers of Mice

**Figure 1:**
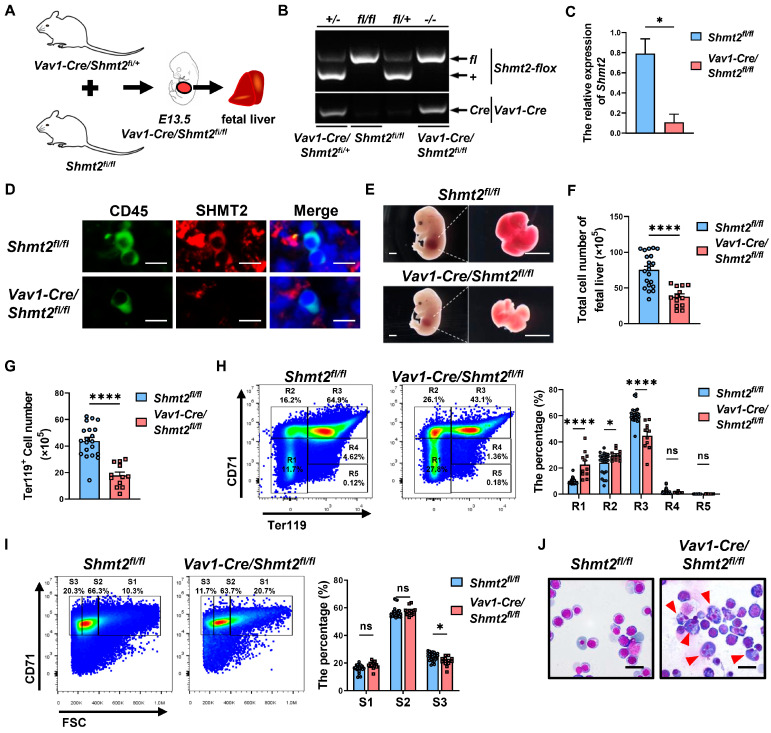
The absence of the *Shmt2* gene within the hematopoietic system impairs the production of erythroid cells in mouse fetal livers. (**A**). Schematic diagram of breeding *Vav1-Cre/Shmt2^fl/fl^* fetal mice with specific knockout of serine hydroxymethyltransferase 2 *(Shmt2)* in the hematopoietic system. (**B**). Agarose gel electrophoresis showing the genotyping results of *Shmt2-flox* (upper) and *Vav1-Cre* (lower) genes. (**C**). RT-qPCR analysis of *Shmt2* expression in CD45^+^ cells derived from fetal livers. Statistics were determined using unpaired Student’s *t*-tests, * *p* < 0.05. *n* = 3. (**D**). Immunofluorescence staining of E13.5 fetal liver cells using anti-CD45 antibody (Alexa Fluor 488; green) and anti-SHMT2 antibody (Alexa Fluor 647; red). Scale bar: 100 μm. (**E**). Images of E13.5 fetal mice and fetal livers captured using a stereomicroscope. Scale bar: 1 mm. (**F**). Cell counting of E13.5 fetal liver cells and comparison of cell numbers between *Shmt2^fl/fl^* (*n* = 21) and *Vav1-Cre/Shmt2^fl/fl^* (*n* = 13) fetal liver cells. Statistics were determined using unpaired Student’s *t*-tests, **** *p* < 0.0001. (**G**). Flow cytometry analysis of the Ter119^+^ cell in fetal livers from *Shmt2^fl/fl^* (*n* = 20) and *Vav1-Cre/Shmt2^fl/fl^* (*n* = 12) embryos. Statistics were determined using unpaired Student’s *t*-tests, **** *p* < 0.0001. (**H**). Representative flow cytometry profiles of R1 to R5 erythroblast populations labeled with CD71 and Ter119 in fetal liver cells at E13.5 (left). The frequency of R1 to R5 cells in fetal livers isolated from *Shmt2^fl/fl^* (*n* = 25) and *Vav1-Cre/Shmt2^fl/fl^* (*n* = 11) embryos (right). Statistics were determined using one-way ANOVA, * *p* < 0.05; **** *p* < 0.0001; ns, not significant. (**I**). Representative flow cytometry profiles of Ter119^hi^ fetal liver cells (E12.5) separated into 3 populations (S1, S2, and S3) based on the forward light scatter (FSC) profile (left). The frequency of S1 to S3 cells in fetal liver erythroid cells isolated from *Shmt2^fl/fl^* (*n* = 25) and *Vav1-Cre/Shmt2^fl/fl^* (*n* = 13) embryos (right). Statistics were determined using one-way ANOVA, * *p* < 0.05; ns, not significant. (**J**). Wright–Giemsa staining of fetal liver cells. The red arrow indicates erythroid precursor cells with irregularly shaped nuclei. Scale bar: 20 μm.

It has been reported that the *Shmt2* gene deletion in mice causes mitochondrial respiratory dysfunction, leading to growth retardation, embryonic lethality, and anemia by Embryonic Day 13.5 (E13.5) [13,14]. However, the possible roles of the *Shmt2* gene on erythopoiesis remain largely unknown. Therefore, we employed advanced gene-editing techniques to generate *Shmt2-flox* mice. These *Shmt2-flox* mice were then crossed with *Vav1-Cre* mice to create a model with hematopoietic cell-specific *Shmt2* deletion [9]. This model allows us to explore the role of SHMT2 in hematopoietic processes (Figure 1A) [10,11,12].

Genotyping confirmed the successful expression of *Vav1-Cre* and *Shmt2-flox* alleles in the mouse cohorts (Figure 1B). Moreover, quantitative gene expression analysis revealed a significant reduction in *Shmt2* expression within CD45^+^ hematopoietic cells isolated from the fetal livers of E13.5 *Vav1-Cre/Shmt2^fl/fl^* mice (Figure 1C). In situ immunofluorescence staining of the fetal livers showed no SHMT2 protein in CD45^+^ hematopoietic cells of *Vav1-Cre/Shmt2^fl/fl^* mice, in contrast to control *Shmt2^fl/fl^* mice (Figure 1D). Collectively, these findings confirm the specific knockout of the Shmt2 gene in the hematopoietic lineage, enabling further investigation into its role in hematopoiesis.

We observed that the fetal livers from *Vav1-Cre/Shmt2^fl/fl^* mice were significantly smaller and paler than those from control mice (Figure 1E). Quantitative cell counts showed a substantial reduction in the total number of fetal liver cells in the knockout group (Figure 1F). Notably, the number of Ter119^+^ erythroid lineage cells was significantly decreased (Figure 1G). These results indicate that SHMT2 deficiency severely affects erythroid development in the fetal liver.

Flow cytometry revealed increased R1 primitive progenitor cells and R2 proerythroblasts cell populations, as well as decreased R3 basophilic erythroblasts in fetal liver erythroid cells from *Vav1-Cre/Shmt2^fl/fl^* mice compared to controls (Figure 1H). A trend toward reduced R4 orthochromatophilic erythroblasts and R5 reticulocytes was observed in the knockout group, though it was not statistically significant, possibly due to small population sizes and variability [15,16]. Additionally, the proportion of smaller S3 cells was significantly reduced in the knockout group (Figure 1I) [17]. Furthermore, Wright–Giemsa staining showed distinct morphological abnormalities in erythroid precursors from *Vav1-Cre/Shmt2^fl/fl^* mice at E13.5. Specifically, these abnormalities included asynchronous development, multinucleation, nuclear budding, and irregular nuclear shapes (Figure 1J) [5]. These characteristics align with megaloblastic anemia, highlighting SHMT2′s critical role in erythroid differentiation [4,6]. Together with flow cytometry results, these findings suggest that SHMT2 deficiency in the hematopoietic system primarily hinders erythroid cell maturation. This aligns with observations of halted erythroid differentiation and maturation in *Shmt2* knockout fetal livers at E13.5 [14].

Overall, our results support the conclusion that the absence of the *Shmt2* gene in the hematopoietic system inhibits the development of erythroid cells in the fetal livers of mice. We speculate that the reduced fetal liver size is attributable to anemia from blocked red blood cell development due to Shmt2 deficiency, thereby impacting liver development.

### 2.2. Anemia-like Symptoms in Adult Mice with the Hematopoietic Cell-Specific Deletion of Shmt2 Gene

**Figure 2:**
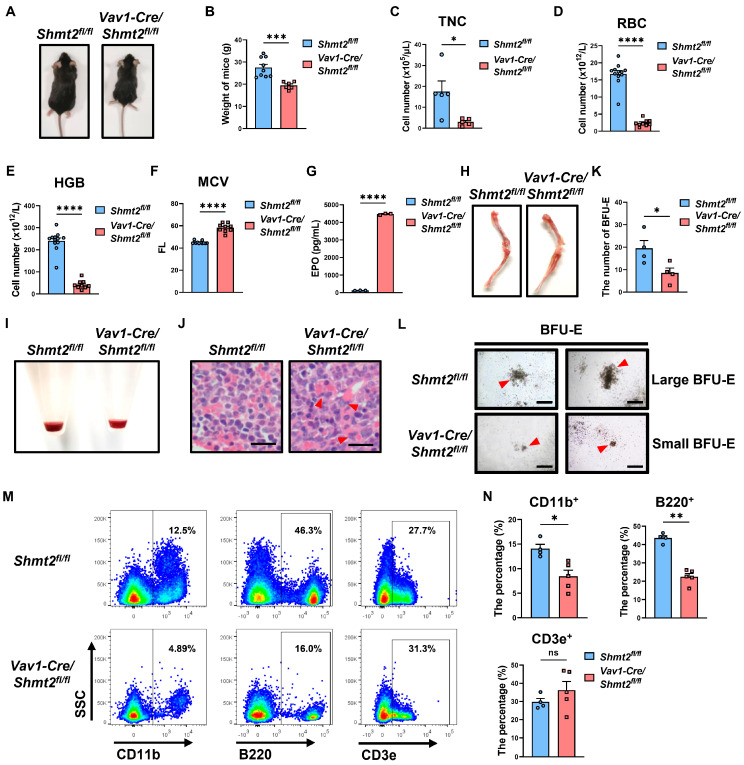
Anemia-like symptoms in adult mice with conditional *Shmt2* deletion. (**A**). Overall view of 10-week-old *Shmt2^fl/fl^* and *Vav1-Cre/Shmt2^fl/fl^* mice. (**B**). Weight comparison between 10-week-old *Shmt2^fl/fl^* (*n* = 9) and *Vav1-Cre/Shmt2^fl/fl^* (*n* = 6) mice. Statistics were determined using unpaired Student’s *t*-tests, *** *p* < 0.001. (**C**). The total nucleated cell (TNC) counts in the peripheral blood of 10-week-old *Shmt2^fl/fl^* (*n* = 5) and *Vav1-Cre/Shmt2^fl/fl^* (*n* = 5) mice were compared. Statistics were determined using unpaired Student’s *t*-tests, * *p* < 0.05. (**D**). red blood cell (RBC) counts in peripheral blood of 10-week-old *Shmt2^fl/fl^* (*n* = 12) and *Vav1-Cre/Shmt2^fl/fl^* (*n* = 9) mice measured by an automatic hematology analyzer. Statistics were determined using unpaired Student’s *t*-tests, **** *p* < 0.0001. (**E**). hemoglobin (HGB) levels in peripheral blood of 10-week-old *Shmt2^fl/fl^* (*n* = 12) and *Vav1-Cre/Shmt2^fl/fl^* (*n* = 9) mice measured by an automatic hematology analyzer. Statistics were determined using unpaired Student’s *t*-tests, **** *p* < 0.0001. (**F**). mean corpuscular volume (MCV) in peripheral blood of 10-week-old *Shmt2^fl/fl^* (*n* = 12) and *Vav1-Cre/Shmt2^fl/fl^* (*n* = 9) mice measured by an automatic hematology analyzer. Statistics were determined using unpaired Student’s *t*-tests, **** *p* < 0.0001. (**G**). Comparison of the erythropoietin (EPO) levels in the peripheral blood serum of 10-week-old *Shmt2^fl/fl^* (*n* = 3) and *Vav1-Cre/Shmt2^fl/fl^* (*n* = 3) mice measured by ELISA. Statistics were determined using unpaired Student’s *t*-tests, **** *p* < 0.0001. (**H**). Images of femurs and tibias from 10-week-old *Shmt2^fl/fl^* and *Vav1-Cre/Shmt2^fl/fl^* mice. (**I**) Precipitates of bone marrow cells from 10-week-old *Shmt2^fl/fl^* and *Vav1-Cre/Shmt2^fl/fl^* mice. (**J**). H&E staining of femur sections from 10-week-old *Shmt2^fl/fl^* and *Vav1-Cre/Shmt2^fl/fl^* mice. Red arrows point to erythroid precursor cells with abnormal nuclei. Scale bar: 20 μm. (**K**). Quantification of burst-forming unit-erythroid (BFU-E) colonies from Methocult cultures of 1 × 10^4^ *Shmt2^fl/fl^* (*n* = 4) and *Vav1-Cre/Shmt2^fl/fl^* (*n* = 4) adult murine bone marrow cells. Statistics were determined using unpaired Student’s *t*-tests, * *p* < 0.05. Scale bar: 500 μm. (**L**). Large (upper) and small (lower) BFU-E colonies from Methocult cultures of 1 × 10^4^ *Shmt2^fl/fl^* and *Vav1-Cre/Shmt2^fl/fl^* adult murine bone marrow cells. Red arrows point to the BFU-E colonies. Scale bar: 500 μm. (**M**). Flow cytometry analysis of CD11b^+^, B220^+^, CD3e^+^ cell in peripheral blood (following erythrocyte lysis) from 10-week-old *Shmt2^fl/fl^* and *Vav1-Cre/Shmt2^fl/fl^* mice. (**N**). The frequencies of CD11b^+^, B220^+^, and CD3e^+^ cells in peripheral blood (following erythrocyte lysis) from 10-week-old *Shmt2^fl/fl^* (*n* = 4) and *Vav1-Cre/Shmt2^fl/fl^* (*n* = 5) mice. Statistics were determined using unpaired Student’s *t*-tests, * *p* < 0.05, ** *p* < 0.01, ns, not significant.

Given that *Shmt2* knockout mice are embryonic lethal, we wonder whether hematopoietic cell-specific *Shmt2*-deficient mice can be born. Interestingly, we observed that, while anemia symptoms manifested in conditional *Shmt2* knockout mice as early as E13.5, these mice were still born and able to survive. We found that the body weight and size of *Vav1-Cre/Shmt2^fl/fl^* mice at 10 weeks were significantly lower than those of the control group (Figure 2A,B). Blood cell counts showed that the number of peripheral blood cells per 10 μL in *Vav1-Cre/Shmt2^fl/fl^* mice was significantly lower than that in the control group (Figure 2C). Hematological assessments showed that both red blood cell (RBC) and hemoglobin (HGB) levels were significantly reduced in the *Vav1-Cre/Shmt2^fl/fl^* group compared to the control mice (Figure 2D,E). In contrast, the mean corpuscular volume (MCV) was significantly higher in *Vav1-Cre/Shmt2^fl/fl^* mice, indicating a greater number of immature and nucleated red blood cells in the peripheral blood (Figure 2F) [4,5]. Additionally, levels of erythropoietin (EPO) in the peripheral blood serum of *Vav1-Cre/Shmt2^fl/fl^* mice were significantly elevated compared to those in control animals, indicating a compensatory response to the anemia (Figure 2G).

Considering that bone marrow (BM) is the main tissue for the maturation of erythroid cells, we further examined the development of erythroid cells in the BM. Notably, we found that the color of the femoral and tibial marrow cavities in the *Vav1-Cre/Shmt2^fl/fl^* group was lighter than that of the control group (Figure 2H), and the color of the BM cell pellets was also significantly lighter (Figure 2I). After H&E staining of femoral bone sections, we observed a significant increase in erythroid precursor cells with segmented and lobulated nuclei in the *Vav1-Cre/Shmt2^fl/fl^* group, suggesting abnormal erythroid development (Figure 2J). When we performed hematopoietic colony-forming assays using the same number of BM cells from the *Vav1-Cre/Shmt2^fl/fl^* group and the control group, we found that the number of burst-forming unit-erythroid (BFU-E) was significantly reduced in the *Vav1-Cre/Shmt2^fl/fl^* group compared to the control mice (Figure 2K). Moreover, under bright-field microscopy, we observed that the size of BFU-E colonies in the *Vav1-Cre/Shmt2^fl/fl^* group was generally smaller than that of the control group (Figure 2L). This observation reinforces our conclusion that SHMT2 significantly compromises the maturation of erythroid cells.

Subsequently, flow cytometric analysis was conducted on T cells (CD3e^+^), myeloid cells (CD11b^+^), and B cells (B220^+^) in the peripheral blood. The results revealed a significant decrease in the proportion of myeloid cells and B cells in the peripheral blood of the *Vav1-Cre/Shmt2^fl/fl^* group, while there was no significant change in T cells (Figure 2M,N). This suggests that the deletion of *Shmt2* in the mouse hematopoietic system not only leads to erythroid developmental arrest, but also impedes the development of myeloid cells and B cells.

In conclusion, our findings suggest that the specific deletion of *Shmt2* in the hematopoietic system leads to prominent anemia-like symptoms in adult mice, likely due to the defect of erythroid development and impaired enucleation processes.

### 2.3. The Deletion of the Shmt2 Gene in the Hematopoietic System Impedes the Enucleation of Red Blood Cells in the Peripheral Blood of Adult Mice

**Figure 3:**
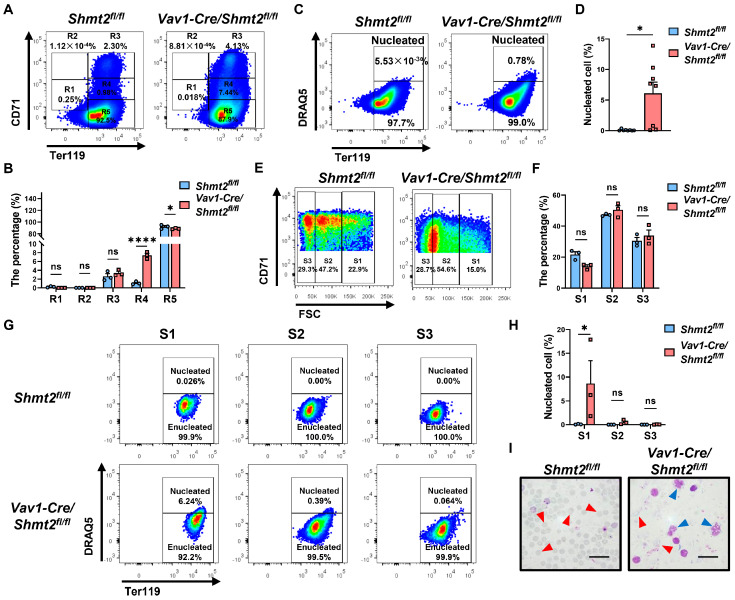
The deletion of the *Shmt2* gene in the hematopoietic system impedes the enucleation of red blood cells in the peripheral blood of adult mice. (**A**). Representative flow cytometry profiles of R1 to R5 erythroblast populations labeled with CD71 and Ter119 in peripheral blood from 10-week-old *Shmt2^fl/fl^* and *Vav1-Cre/Shmt2^fl/fl^* mice. (**B**). Frequency of R1 to R5 cells in peripheral blood from 10-week-old *Shmt2^fl/fl^* (*n* = 3) and *Vav1-Cre/Shmt2^fl/fl^* (*n* = 3) mice. Statistics were determined using one-way ANOVA, * *p* < 0.05; **** *p* < 0.0001; ns, not significant. (**C**). Flow cytometry analysis of DRAQ5^+^ nucleated cells in Ter119^hi^ peripheral blood cells from 10-week-old *Shmt2^fl/fl^* and *Vav1-Cre/Shmt2^fl/fl^* mice. (**D**). The frequency of DRAQ5^+^ nucleated cells in Ter119^hi^ peripheral blood cells from 10-week-old Shmt2^fl/fl^ (*n* = 8) and Vav1-Cre/Shmt2^fl/fl^ (*n* = 8) mice. Statistics were determined using unpaired Student’s *t*-tests, * *p* < 0.05. (**E**). Representative flow cytograms of Ter119^hi^ peripheral blood cells separated into 3 populations (S1, S2, and S3) based on the forward light scatter (FSC) profile. (**F**). The frequency of S1 to S3 cells in peripheral blood from 10-week-old *Shmt2^fl/fl^* (*n* = 3) and *Vav1-Cre/Shmt2^fl/fl^* (*n* = 3) mice. Statistics were determined using one-way ANOVA; ns, not significant. (**G**). Flow cytometry analysis of Ter119^hi^ DRAQ5^+^ nucleated cells in peripheral blood S1 to S3 cells from 10-week-old *Shmt2^fl/fl^* and *Vav1-Cre/Shmt2^fl/fl^* mice. (**H**). The frequency of DRAQ5^+^ nucleated cells in peripheral blood S1 to S3 cells from 10-week-old *Shmt2^fl/fl^* (*n* = 3) and *Vav1-Cre/Shmt2^fl/fl^* (*n* = 3) mice. Statistics were determined using one-way ANOVA, * *p* < 0.05; ns, not significant. (**I**). Wright–Giemsa staining of peripheral blood cells from 10-week-old *Shmt2^fl/fl^* and *Vav1-Cre/Shmt2^fl/fl^* mice. Red arrows indicate enucleated erythrocytes; blue arrows indicate nucleated erythrocytes. Scale bar: 20 μm.

Our data indicated that significant defects in the late stages of erythroblast maturation following the deletion of the *Shmt2* gene in the hematopoietic system. In support of this, flow cytometry analysis of peripheral blood cells of these adult mice revealed a significantly higher proportion of immature R4 orthochromatophilic erythroblasts and a lower proportion of more mature R5 reticulocytes in *Vav1-Cre/Shmt2^fl/fl^* mice compared to the control group (Figure 3A,B) [15,16]. Flow cytometric evaluations of the enucleation rate of Ter119^hi^ erythroid cells in the peripheral blood showed a markedly higher proportion of nucleated cells in the *Vav1-Cre/Shmt2^fl/fl^* group compared to the control group (Figure 3C,D). By combining the expression levels of Ter119^hi^ cells with forward light scatter and CD71, we classified the cells into three subpopulations: S1 (large, nucleated cells), S2 (medium-sized, early enucleating cells), and S3 (small, enucleated cells), representing the progressive maturation of red blood cells [17]. Statistical analysis of the proportions of these subpopulations showed no significant difference in the S1, S2, and S3 cell populations between the *Vav1-Cre/Shmt2^fl/fl^* group and the control group (Figure 3E,F). However, further staining with the nuclear dye DRAQ5 revealed a significantly higher proportion of nucleated erythroblasts in the *Vav1-Cre/Shmt2^fl/fl^* group compared to the control group, primarily occurring in the larger S1 subpopulation (Figure 3G,H). Wright–Giemsa staining of peripheral blood smears revealed an increased number of immature nucleated red blood cells in *Vav1-Cre/Shmt2^fl/fl^* mice (Figure 3I). These results indicate a significant block in the enucleation of erythroid cells during the early stages of terminal erythroid maturation.

### 2.4. The Absence of the Shmt2 Gene in the Hematopoietic System Hinders the Development of Erythroid Cells in the Bone Marrow of Adult Mice

**Figure 4:**
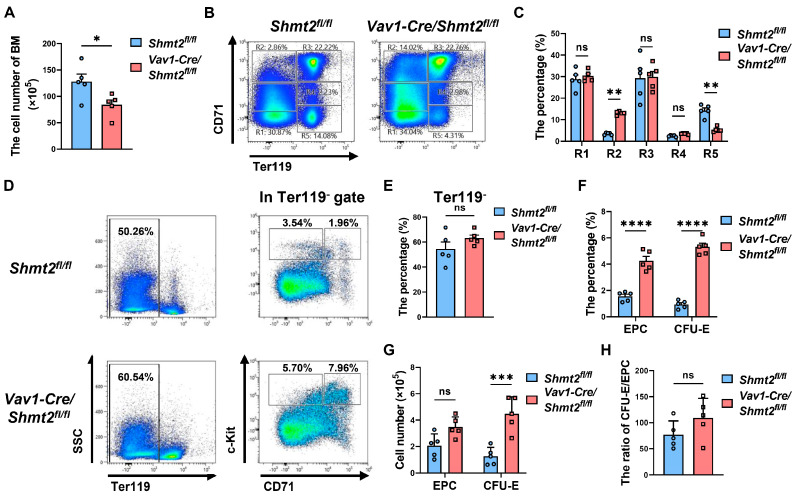
The absence of *Shmt2* gene in the hematopoietic system hinders the development of erythroid cells in the bone marrow of adult mice. (**A**). BM cells counts from 10-week-old *Shmt2^fl/fl^* (*n* = 5) and *Vav1-Cre/Shmt2^fl/fl^* (*n* = 5) mice were compared. Statistics were determined using unpaired Student’s *t*-tests, * *p* < 0.05. (**B**). Representative flow cytometry profiles of R1 to R5 erythroblast populations labeled with CD71 and Ter119 in BM from 10-week-old adult mice. (**C**). The frequency of R1 to R5 cells in BM from *Shmt2^fl/fl^* (*n* = 5) and *Vav1-Cre/Shmt2^fl/fl^* (*n* = 5) 10-week-old adult mice. Statistics were determined using one-way ANOVA, ** *p* < 0.01; ns, not significant. (**D**). Representative flow cytometric results of the expression of surface markers Ter119, CD43, CD71, and c-Kit in BM cells from 10-week-old adult mice. (**E**,**F**). The bar graph shows the percentages of Ter119^−^ cells, early progenitor cells (EPC, Ter119^−^ CD71^−^ c-Kit^+^), and colony-forming unit-erythroid (CFU-E, Ter119^−^ CD71^+^ c-Kit^+^) cells derived from the BM cells of 10-week-old *Shmt2^fl/fl^* (*n* = 5) and *Vav1-Cre/Shmt2^fl/fl^* (*n* = 5) adult mice; Statistics were determined using unpaired Student’s *t*-tests, **** *p* < 0.0001; ns, not significant. (**G**). The bar graph shows the cell numbers of EPC (Ter119^−^ CD71^−^ c-Kit^+^) cells and CFU-E (Ter119^−^ CD71^+^ c-Kit^+^) cells derived from the BM cells of 10-week-old *Shmt2^fl/fl^* (*n* = 5) and *Vav1-Cre/Shmt2^fl/fl^* (*n* = 5) adult mice. Statistics were determined using unpaired Student’s *t*-tests, *** *p* < 0.001; ns, not significant. (**H**). The bar graph shows the ratio of CFU-E/EPC. Statistics were determined using unpaired Student’s *t*-tests, ns, not significant.

Through examination of peripheral blood, we know that the loss of SHMT2 affects the terminal maturation and denucleation of erythroid cells, but at which stage of erythroid development does SHMT2 specifically exert its influence? To answer this question, we isolated BM cells from the *Vav1-Cre/Shmt2^fl/fl^* group and the control group and found that the *Vav1-Cre/Shmt2^fl/fl^* group had fewer cells (Figure 4A). Flow cytometry analysis of erythroid cells revealed that the proportion of proerythroblasts (R2 cell population) was significantly higher in the *Vav1-Cre/Shmt2^fl/fl^* group compared to the control group, while there was no significant difference in the basophilic erythroblasts (R3 cell population). The reticulocyte (R5 cell population) was significantly lower in the *Vav1-Cre/Shmt2^fl/fl^* group, indicating a clear erythroid developmental arrest in adult mice due to the loss of *Shmt2* (Figure 4B,C). Since the increase in the proportion mainly occurred in the proerythroblast population, we further analyzed the early progenitor cell (EPC, Ter119^−^ CD71^-^ c-Kit^+^) and colony-forming unit-erythroid (CFU-E) (Ter119^−^ CD71^+^ c-Kit^+^) populations according to the expression of Ter119 for mature erythroid cells, CD71 for the early erythroid cells, and c-Kit for the hematopoietic cells (Figure 4D) [18,19]. The results showed that there was no significant difference in the proportion of Ter119^−^ cells between the *Vav1-Cre/Shmt2^fl/fl^* group and the control group. Notably, the proportions of early progenitor and CFU-E cells were both higher in the *Vav1-Cre/Shmt2^fl/fl^* group, and the number of CFU-E cells was also higher in the *Vav1-Cre/Shmt2^fl/fl^* group (Figure 4E–G) However, when we further compared the ratio of CFU-E cells to early progenitor cells, we found that the *Vav1-Cre/Shmt2^fl/fl^* group had no significant differences from the control group (Figure 4H), indicating that the deficiency of *Shmt2* showed no obvious developmental disorders of early erythroid cells, including BFU-E and CFU-E. As the proportion of downstream basophilic erythroblasts remained unchanged, all of the above results suggest that erythroid developmental arrest likely occurs during the development of CD71^+^ Ter119^+^ cells.

**Figure 5:**
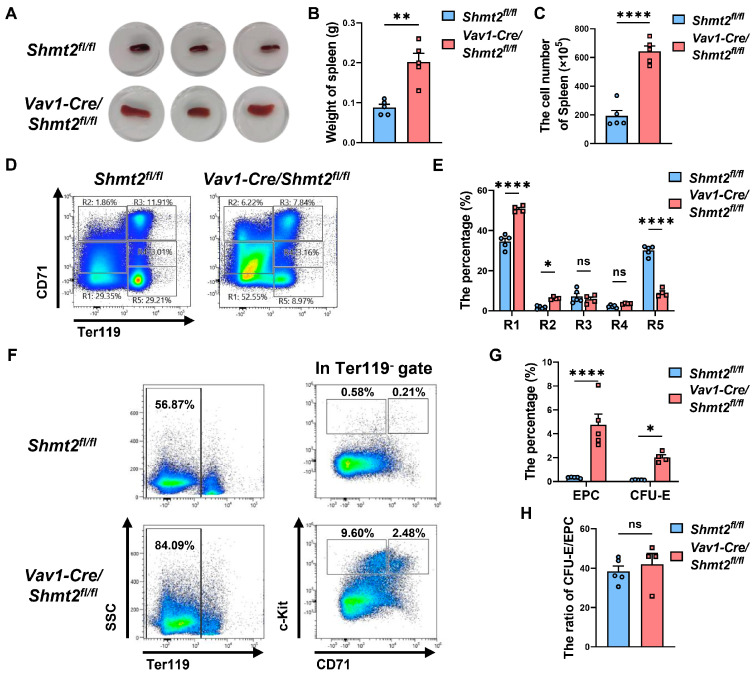
The absence of the *Shmt2* gene in the hematopoietic system results in splenic abnormalities in adult mice. (**A**). Spleen images of 10-week-old *Shmt2^fl/fl^* and *Vav1-Cre/Shmt2^fl/fl^* mice. (**B**). The spleens of 10-week-old *Shmt2^fl/fl^* and *Vav1-Cre/Shmt2^fl/fl^* mice were weighed, and the difference in weight between *Shmt2^fl/fl^* (*n* = 5) and *Vav1-Cre/Shmt2^fl/fl^* (*n* = 5) mice spleens was compared. Statistics were determined using unpaired Student’s *t*-tests, ** *p* < 0.01. (**C**). Cell counts were performed on spleen cells from 10-week-old adult mice, and the difference in spleen cell numbers between *Shmt2^fl/fl^* (*n* = 5) and *Vav1-Cre/Shmt2^fl/fl^* (*n* = 5) mice was compared. Statistics were determined using unpaired Student’s *t*-tests, **** *p* < 0.0001. (**D**). Representative flow cytometry profiles of R1 to R5 erythroblast populations labeled with CD71 and Ter119 in the spleens of 10-week-old adult mice. (**E**). The frequency of R1 to R5 cells in the spleens of *Shmt2^fl/fl^* (*n* = 5) and *Vav1-Cre/Shmt2^fl/fl^* (*n* = 5) 10-week-old adult mice. Statistics were determined using one-way ANOVA, * *p* < 0.05; **** *p* < 0.0001; ns, not significant. (**F**). Representative flow cytometry results of surface markers Ter119, CD43, CD71, and c-Kit in spleen cells from 10-week-old adult mice. (**G**). The bar graph shows the percentage of EPC (Ter119^−^ CD71^−^ c-Kit^+^) cells and CFU-E (Ter119^−^ CD71^+^ c-Kit^+^) cells derived from the spleen cells of *Shmt2^fl/fl^* (*n* = 5) and *Vav1-Cre/Shmt2^fl/fl^* (*n* = 5) 10-week-old adult mice. Statistics were determined using unpaired Student’s *t*-tests, * *p* < 0.05; **** *p* < 0.0001. (**H**). The bar graph shows the ratio of CFU-E/EPC. Statistics were determined using unpaired Student’s *t*-tests, ns, not significant.

Subsequently, we observed the spleens of *Vav1-Cre/Shmt2^fl/fl^* mice and control mice and found that the spleens of *Vav1-Cre/Shmt2^fl/fl^* mice were significantly larger (Figure 5A) and heavier (Figure 5B) compared to the control group. Cell counting of splenic cells revealed that the number of splenic cells in *Vav1-Cre/Shmt2^fl/fl^* mice was significantly higher than in the control group (Figure 5C), consistent with the phenomenon of compensatory splenic enlargement caused by anemia. By marking erythroid cells with surface markers CD71 and Ter119, the flow cytometry results showed that, similar to the bone marrow findings, the proportions of primitive progenitor cells (R1 cell population) and proerythroblasts (R2 cell population) in the spleens of *Vav1-Cre/Shmt2^fl/fl^* mice were significantly higher than those in the control group, while there was no significant difference in the proportion of basophilic erythroblast (R3 cell population). However, the reticulocyte (R5 cell population) proportion was significantly lower than in the control group (Figure 5D,E). Further examination of early progenitor cells and CFU-E cells showed that the proportions of early progenitor cells and CFU-E cells in the *Vav1-Cre/Shmt2^fl/fl^* group were both higher than in the control group (Figure 5F,G). Comparing the proportion of the CFU-E cell population to that of the early progenitor cell population, we found no significant difference between the *Vav1-Cre/Shmt2^fl/fl^* group and the control group (Figure 5H). Collectively, these results substantiate our conclusion that the impairment in erythroid development and maturation resulting from SHMT2 deficiency primarily occurs during the basophilic erythroblast developmental stage. This defect facilitates the release of large numbers of immature nucleated erythrocytes and erythroid progenitor cells into circulation, explaining the elevated levels of nucleated erythrocytes observed in the peripheral blood and the increased erythroid progenitor cells in the spleen.

### 2.5. Promotion of Erythroid-Related Gene Expression by Shmt2 Deletion in Proerythroblasts

**Figure 6:**
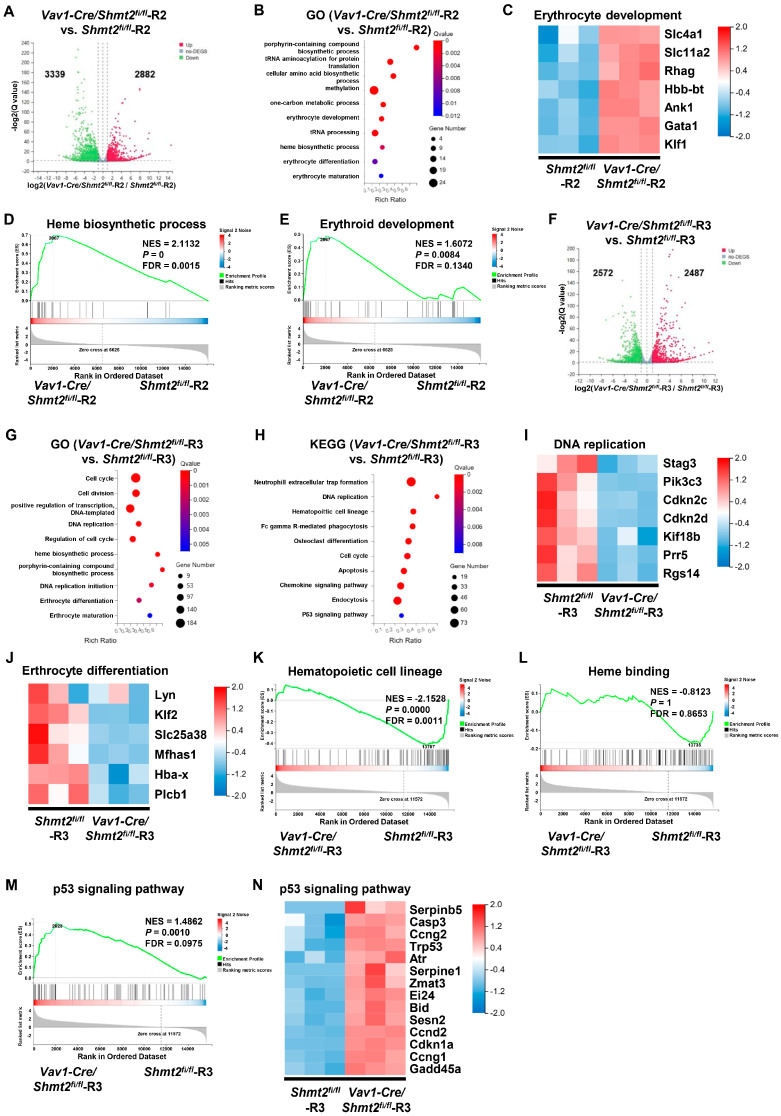
The deletion of the *Shmt2* gene impedes the proliferation and erythroid differentiation of orthochromatic erythroblasts in mouse bone marrow. (**A**). Volcano plot showing significantly changed genes between *Vav1-Cre/Shmt2^fl/fl^*-R2 and *Shmt2^fl/fl^*-R2. Red dots represent upregulated genes. Green dots represent downregulated genes. Gray dots represent genes that were not differentially expressed (*p* < 0.05, |logFC| > 1.0). (**B**). Gene Ontology (GO) enrichment analysis revealed the top terms enriched by the upregulated genes in *Vav1-Cre/Shmt2^fl/fl^*-R2 compared to *Shmt2^fl/fl^*-R2. The size of each dot is based on the number of genes enriched in the pathway, and the color of the dots represents the significance of the pathway enrichment. (**C**). Heatmap illustrating the relative expression of the RNA-seq data of erythrocyte development-related genes. (**D**). Gene Set Enrichment Analysis (GSEA) comparing *Vav1-Cre/Shmt2^fl/fl^*-R2 and *Shmt2^fl/fl^*-R2 for heme biosynthetic process differentiation gene sets. (**E**). GSEA comparing *Vav1-Cre/Shmt2^fl/fl^*-R2 and *Shmt2^fl/fl^*-R2 for erythrocyte development differentiation gene sets. (**F**). Volcano plot showing significantly changed genes between *Vav1-Cre/Shmt2^fl/fl^*-R3 and *Shmt2^fl/fl^*-R3. Red dots represent upregulated genes. Green dots represent downregulated genes. Gray dots represent genes that were not differentially expressed (*p* < 0.05, |logFC| > 1.0). (**G**). GO enrichment analysis revealed the top terms enriched by the upregulated genes in *Vav1-Cre/Shmt2^fl/fl^*-R3 compared to *Shmt2^fl/fl^*-R3. The size of each dot is based on the number of genes enriched in the pathway, and the color of the dots represents the significance of pathway enrichment. (**H**). Kyoto Encyclopedia of Genes and Genomes (KEGG) enrichment analysis revealed the top terms enriched by the upregulated genes in *Vav1-Cre/Shmt2^fl/fl^*-R3 compared to *Shmt2^fl/fl^*-R3. The size of each dot is based on the number of genes enriched in the pathway, and the color of the dots represents the significance of pathway enrichment. (**I**). Heatmap illustrating the relative expression of the RNA-seq data of DNA replication-related genes. (**J**). Heatmap illustrating the relative expression of the RNA-seq data of erthrocyte differentiation-related genes. (**K**). GSEA comparing *Vav1-Cre/Shmt2^fl/fl^*-R3 and *Shmt2^fl/fl^*-R3 for hematopoietic cell lineage differentiation gene sets. (**L**). GSEA comparing *Vav1-Cre/Shmt2^fl/fl^*-R3 and *Shmt2^fl/fl^*-R3 for heme binding differentiation gene sets. (**M**). GSEA comparing *Vav1-Cre/Shmt2^fl/fl^*-R3 and *Shmt2^fl/fl^*-R3 for p53 signaling pathway differentiation gene sets. (**N**). Heatmap illustrating the relative expression of the RNA-seq data of p53 signaling pathway-related genes.

To further investigate the role of SHMT2 in erythroid differentiation, we isolated CD71^+^ Ter119^−^ (R2 cell population) cells from the bone marrow of *Vav1-Cre/Shmt2^fl/fl^* mice and *Shmt2^fl/fl^* mice for transcriptome sequencing. We found that there were 2882 upregulated genes and 3339 downregulated genes in the CD71^+^ Ter119^−^ cells of the *Vav1-Cre/Shmt2^fl/fl^* group compared to the control group, indicating a significant impact of *Shmt2* deletion on the gene expression of erythroid precursor cells (Figure 6A). Given the increased proportion of the R2 cell population in the *Shmt2*-deficient mice, we speculated that the deletion of *Shmt2* in the hematopoietic system would promote erythroid differentiation in the R2 cell population. Analysis of significantly enriched Gene Ontology (GO) terms revealed that the upregulated genes in the *Vav1-Cre/Shmt2^fl/fl^* group were enriched in erythroid development-related pathways such as porphyrin-containing compound biosynthesis, erythrocyte development, heme biosynthesis, and erythrocyte differentiation. Additionally, we also observed enrichment of upregulated genes in methylation and one-carbon metabolism processes (Figure 6B). These significantly changed genes enriched in erythroid differentiation pathways are displayed in the heatmap, including the gene encoding adult hemoglobin β-polypeptide chain *Hbb-bt*; genes promoting erythroid development *Ank1*, *Gata1*, *Klf1*, *Slc4a1*, *Slc11a2*; and the erythrocyte function-related gene *Rhag* (Figure 6C). Gene Set Enrichment Analysis (GSEA) further revealed that most of the differentially expressed genes upregulated in the *Vav1-Cre/Shmt2^fl/fl^* group were enriched in heme biosynthesis and erythroid development pathways (Figure 6D,E). These results confirm our speculation that the deletion of *Shmt2* in the hematopoietic system promotes erythroid differentiation in the R2 cell population.

### 2.6. Impaired Proliferation and Erythroid Differentiation in Basophilic Erythroblasts Due to Shmt2 Deletion

Transcriptome sequencing analysis of CD71^+^ Ter119^+^ (R3 cell population) cells revealed that there were 2487 upregulated genes and 2572 downregulated genes in the *Vav1-Cre/Shmt2^fl/fl^* group compared to the control group (Figure 6F). Subsequently, GO enrichment analysis of differentially expressed genes (FC > 1) revealed that the downregulated genes were significantly enriched in cell cycle, cell division, positive regulation of transcription, and DNA replication, indicating that the loss of *Shmt2* may significantly inhibit the proliferation of CD71^+^ Ter119^+^ cells. Concurrently, we found that the downregulated genes were also enriched in heme biosynthesis, porphyrin-containing compound biosynthesis, erythroid differentiation, and maturation pathways, suggesting that the deletion of *Shmt2* may significantly inhibit the development of CD71^+^ Ter119^+^ cells into mature erythroid cells (Figure 6G). Kyoto Encyclopedia of Genes and Genomes (KEGG) enrichment analysis of downregulated genes also revealed enrichment in DNA replication, cell cycle, cell apoptosis, and p53 signaling pathways, consistent with the GO analysis, indicating that the loss of *Shmt2* may significantly inhibit the proliferation of CD71^+^ Ter119^+^ cells. The enrichment of p53 pathways may suggest that the deletion of *Shmt2* leads to apoptosis in CD71^+^ Ter119^+^ cells (Figure 6H). When displaying the differentially expressed genes related to DNA replication in a heatmap, we observed significant decreases in the transcriptional levels of *Cdkn2c*, *Cdkn2d*, which are closely related to the regulation of the cell cycle G1 phase; *Stag3*, *Pik3c3*, *Kif18b*, *Rgs14*, which are involved in cell division; and *Prr5*, which acts as a mTORC2 subunit and participates in cell growth, in CD71^+^ Ter119^+^ cells from the *Vav1-Cre/Shmt2^fl/fl^* group (Figure 6I). Simultaneously, we found significant decreases in the transcriptional levels of *Hba-x*, *Plcb1*, *Lyn*, and *Klf2*, which play crucial roles in erythrocyte maturation; *Mfhas1*, which participates in erythrocyte differentiation by activating the ERK1/ERK2 signaling pathway; and *Slc25a38*, a membrane protein involved in erythrocyte differentiation, in CD71^+^ Ter119^+^ cells from the *Vav1-Cre/Shmt2^fl/fl^* group (Figure 6J). GSEA also revealed that most of the downregulated differentially expressed genes in the *Vav1-Cre/Shmt2^fl/fl^* group were enriched in hematopoietic cell lineages and heme binding (Figure 6K,L). The results of transcriptome sequencing indicate that the deletion of the *Shmt2* gene impairs the proliferation and erythroid differentiation capacity of CD71^+^ Ter119^+^ cells. Additionally, to further unravel the mechanism of erythroid arrest by *Shmt2* deficiency, we found that the upregulated differentially expressed genes in the *Vav1-Cre/Shmt2^fl/fl^* group were enriched in the p53 signaling pathway (Figure 6M). Meanwhile, the transcriptional levels of *Serpinb5* and *Sesn2*, direct targets of the p53-related pathway; apoptosis-related genes such as *Casp3*, *Ei24*, *Bid*, *Serpine1*, and *Zmat3*; *Ccng2*, *Ccnd2*, and *Atr*, which are negatively related to cell cycle regulation; and *Cdkn1a*, *Ccng1*, *Gadd45a*, and *Trp53*, which participate in p53/TP53-mediated inhibition of cell proliferation, were significantly increased in CD71^+^ Ter119^+^ cells from the *Vav1-Cre/Shmt2^fl/fl^* group (Figure 6N). This pathologic p53 activation is most likely the main reason for preventing normal expansion of erythroid progenitor cells.

These findings validate our hypothesis that the deletion of the *Shmt2* gene promotes erythroid development in the R2 cell population while impeding the proliferation and erythroid differentiation of the R3 cell population in mouse bone marrow, which is most likely due to the pathological p53 activation, leading to an increased proportion of R2 cells and erythroid developmental arrest in the R3 cell population. This dual effect of *Shmt2* deletion underscores its essential role in regulating both the differentiation and proliferation of erythroid cells in bone marrow.

## 3. Discussion

Anemia is defined as a pathological condition characterized by a reduction in either the number of circulating RBCs or the hemoglobin concentration in comparison to age-matched controls [20]. In 2021, the global prevalence of anemia in all age groups was approximately 24.3%, corresponding to 1.92 billion (1.89–1.95) prevalent cases [21]. This condition is associated with numerous adverse health outcomes, including increased morbidity and mortality, as well as significant healthcare costs, thereby constituting a considerable threat to public health [22].

The investigation of the pathogenic mechanisms underlying anemia is of paramount importance for the development of effective therapeutic strategies. In this study, we focus on SHMT2, a pivotal enzyme in serine and folate metabolism, and its role in erythroid development within the murine hematopoietic system. SHMT2 catalyzes the transfer of a hydroxymethyl group from serine to tetrahydrofolate, yielding N^5^N^10^-methylenetetrahydrofolate and glycine while also generating NADPH and a methyl group for S-adenosylmethionine synthesis [7,8]. This enzyme is instrumental to various biological processes, including serine metabolism, one-carbon metabolism, tRNA synthesis, and the regulation of epigenetic modifications [23]. Notably, studies have demonstrated that SHMT2 facilitates the conversion of serine to glycine, thereby producing active one-carbon units that are crucial for DNA and histone methylation, processes that can potentially initiate lymphomagenesis through the silencing of tumor suppressor genes [24].

Preliminary investigations conducted in our laboratory have indicated that serine utilization is particularly pronounced during the erythroid differentiation of hematopoietic progenitor cells derived from umbilical cord blood. In a serine-deficient culture system, we observed significant inhibition of cell proliferation and reduced viability of the human umbilical cord blood-derived erythroid progenitor-2 (HUDEP-2) erythroid cell line, suggesting that serine metabolism plays a critical role in regulating the proliferation and differentiation of stem cells.

To elucidate the specific contributions of SHMT2 to erythroid development, we constructed *Shmt2-flox* homozygous mice using the Cre-lox recombination system and bred them with *Vav1-Cre* mice, successfully establishing a *Vav1-Cre/Shmt2^fl/fl^* mouse model that selectively knocks out *Shmt2* within the hematopoietic system. Based on this model, we noted that the fetal livers of *Vav1-Cre/Shmt2^fl/fl^* conditional knockout mice exhibited a significant reduction in size and a lighter coloration compared to control mice.

Flow cytometric analysis revealed an increased proportion of R1 primitive progenitor cells and R2 proerythroblasts, accompanied by a marked decrease in R3 basophilic erythroblasts, suggesting that the deletion of the *Shmt2* gene disrupts erythroid development within the fetal liver. The observed reduction in fetal liver size is likely attributable to impaired erythroid development resulting from SHMT2 deficiency, which in turn could lead to anemia and compromise normal fetal liver development without inducing embryonic lethality. This observation provides a foundation for further investigation into SHMT2′s role in adult murine hematopoietic systems.

Subsequent analyses indicated that the specific deletion of *Shmt2* in the hematopoietic system leads to a significant anemia phenotype in the peripheral blood of adult mice, which may be caused by immature erythroid development. Furthermore, we observed a significant block in erythroid cell denucleation during the early stage of terminal differentiation of erythrocytes. To further confirm the occurrence of erythroid development blockage, we performed flow cytometry and hematopoietic colony-forming experiments on erythroid cells from the BM of adult mice. It was found that the deletion of *Shmt2* does not affect the development erythroid progenitor cells, but significantly affects the maturation of erythroid cells. This maturation blockage mainly occurs during the development of basophilic erythroblasts. At the same time, we found that *Vav1-Cre/Shmt2^fl/fl^* conditional knockout mice also exhibited significant splenomegaly, which is often caused by anemia [25,26,27]. Considering flow cytometry data from bone marrow, splenocytes, and peripheral blood, we propose that the entry of immature nucleated red blood cells and erythroid progenitors from the bone marrow into circulation accounts for their increased presence in the periphery and spleen.

To further validate our findings and investigate the underlying mechanisms responsible for this phenomenon, we conducted transcriptome sequencing and analysis of the R2 and R3 cell population in the BM of *Vav1-Cre/Shmt2^fl/fl^* group mice and control group mice through flow cytometry sorting. Our results indicated that the absence of *Shmt2* in the hematopoietic system led to an upregulation of genes related to erythroid development in the R2 cell population, which promoted erythroid differentiation. Conversely, this deficiency resulted in a significant downregulation of erythroid development-related genes and DNA replication-related genes in the R3 cell population, thus impeding the proliferation and erythroid differentiation ability of the R3 cell population in mouse bone marrow. Furthermore, we observed that SHMT2 deficiency was associated with the upregulation of the p53 signaling pathway and its related genes in the R3 cell population, which contributed to an increased proportion of R2 cells and a blockade in erythroid development within the R3 population. This suggests that SHMT2 may regulate erythroid differentiation by modulating p53-dependent pathways, which govern cell cycle arrest, apoptosis, and DNA damage responses across various stages of erythroid development [28,29].

## 4. Materials and Methods

### 4.1. Mice

To generate *Shmt2-flox* mice on a C57BL/6N background, we employed the Cas9/sgRNA injection method into fertilized eggs. The sgRNA sequence designed for the 5′ target site is: 5′-ATGGAGGTATTCATGTCCGGAGG-3′; and the sgRNA sequence for the 3′ target site is: 5′-ATGAACGTTGAGGACAGTTGAGG-3′. The resulting F0 generation mice may be mosaic/heterozygous/homozygous, and by mating F0 mice with wild-type mice, we obtained the F1 generation. After genotyping and sequencing, we identified stably inherited *Shmt2-flox* mice. To achieve hematopoietic cell-specific deletion of the target gene, we crossed floxed heterozygous mice with *Vav1-Cre* mice [11,30]. All mice were maintained in specific pathogen-free conditions in our institute’s animal facility. All animal experiments were reviewed and approved by our institutional animal care and use committee.

### 4.2. Blood Routine Test

Using a fully automatic hematology analyzer (Nihon Kohden, Shanghai, China), we conducted complete blood count tests on mice to measure various hematological parameters, including RBC count, HGB concentration, and MCV.

### 4.3. Determination of Serum EPO by ELISA

To determine the level of EPO in mouse serum using a commercial kit (EK0333, BOSTER, Wuhan, China), blood was collected via retro-orbital puncture and allowed to clot at room temperature for 2 h. Subsequently, the serum was obtained by centrifuging at 2000× *g* for 20 min, and ELISA was performed immediately following the kit’s instructions.

### 4.4. Quantitative Real-Time PCR

Total RNA was isolated using the TRIzol reagent (ThermoFisher, Carlsbad, CA, USA). And RNA was reverse-transcripted into cDNA using a qPCR RT Kit (TOYOBO, Shanghai, China). qRT-PCR was performed using SYBR Green PCR Master Mix (Bio-Rad, Hercules, CA, USA) in Bio-Rad iQ5 (Bio-Rad) and analyzed by Bio-Rad CXF Manager software (Version 3.1, Bio-Rad, Hercules, CA, USA). The gene expression levels were normalized to the housekeeping gene *Actb*. The primer sequences were obtained from PrimerBank (https://pga.mgh.harvard.edu/primerbank/, accessed on 21 November 2022). The primer sequences used in qRT-PCR analysis are listed in Supplementary Table S1.

### 4.5. Immunofluorescence Staining

For immunofluorescence staining (IF), cells were fixed with 4% paraformaldehyde, permeabilized with 0.2% Triton X-100, and treated with 2% donkey serum, and the antibody was incubated. Images were taken by a Zeiss inverted microscope. The antibodies used in immunofluorescence staining are listed in Supplementary Table S2.

### 4.6. Flow Cytometry Analysis

After performing cell counting using a cell viability analyzer, 100 µL of PBS was used to resuspend 10^6^ cells as a sample. Following the manufacturer’s instructions, an appropriate amount of flow cytometry antibodies was added to each tube of cells and incubated for 1 h at 4 °C. Due to the limited number of cells at each stage, which was caused by stepwise induced differentiation, compensation beads (Anti-Mouse Ig, κ/Negative Control (BSA) Compensation Plus (7.5 µm) Particles Set) were used to individually label antibodies with different fluorophores in lieu of cells to adjust the fluorescence compensation. Additionally, a blank control group without antibody labeling was set up and also incubated for 1 h at 4 °C. After the incubation, the cells are washed twice with PBS, resuspended in an appropriate amount of PBS, and then analyzed using a flow cytometer. Three replicates were set up for each group. Flow cytometric analysis was performed using the Guava easyCyte flow cytometer (Luminex, Austin, TX, USA). The antibodies used in flow cytometry analysis are listed in Supplementary Table S2.

### 4.7. Colony-Forming Unit Assay

For the hematopoietic colony-forming unit (CFU) assay, the precipitated cells were diluted to 2 × 10^5^ cells/mL using IMDM medium. We mixed 150 μL of cell suspension with 1.5 mL of MethoCult M3434 (03434, STEMCELL Technologies, Shanghai, China) and then seeded 1 mL per well in a six-well plate. The mixture was incubated in a CO_2_ incubator for 14 days. The resulting colonies could be classified into burst-forming unit-erythroid (BFU-E), colony-forming unit–granulocyte–macrophage (CFU-GM), and colony-forming unit–granulocyte–erythroid–macrophage–megakaryocyte (CFU-GEMM).

### 4.8. Wright-Giemsa Staining

The cell concentration was adjusted to 1 × 10^6^ cells/mL using PBS. A 100 μL aliquot of the cell suspension was dropped onto a glass slide to create a cell smear. The smear was then left to dry in a biosafety cabinet. After drying, the A solution of the Wright–Giemsa stain kit (BA4017, BASO, Zhuhai, China) was applied to the cell area and allowed to react for 1 min. Subsequently, a volume of B solution was added and allowed to react for 9 min. The stain was then rinsed off with running water. The stained smear was photographed using an oil immersion lens on a brightfield microscope (Olympus, Tokyo, Japan).

### 4.9. RNA Isolation and Library Preparation

Total RNA was extracted using the TRIzol reagent according to the manufacturer’s protocol. mRNA enrichment was performed by using Oligo dT magnetic beads to enrich polyA-tailed mRNA. The obtained RNA was fragmented using a fragmentation buffer, and then reverse transcription was carried out with random N6 primers to synthesize the first-strand cDNA. Subsequently, the second-strand cDNA was synthesized to form double-stranded DNA. The synthesized double-stranded DNA was end-repaired and phosphorylated at the 5′ end, with a protruding ‘A’ base formed at the 3′ end. A bubble-shaped adapter with a protruding ‘T’ base at the 3′ end was then ligated. The ligation product was amplified by PCR using specific primers. The PCR product was thermally denatured into single-stranded DNA, and then a bridge primer was used to circularize the single-stranded DNA to obtain a single-stranded circular DNA library. After quality inspection of the constructed library, sequencing was performed if the quality met the requirements.

### 4.10. RNA Sequencing and Differentially Expressed Genes Analysis

The sequencing process was conducted using the DNBSEQ platform with PE100 (read length). The data obtained from sequencing were referred to as raw reads or raw data. Subsequently, quality control (QC) was performed on the raw reads to determine whether the sequencing data were suitable for further analysis. After QC, the filtered clean reads were aligned to a reference sequence. Following the alignment, the alignment results were evaluated by assessing the alignment rate and the distribution of reads on the reference sequence to determine whether they passed the second quality control assessment (QC of alignment). If the results met the criteria, gene quantification analysis and various analyses based on gene expression levels (such as principal component analysis, correlation analysis, differential gene screening, etc.) were conducted. Additionally, deeper mining analyses such as GO and KEGG functional enrichment analysis, pathway enrichment analysis, and clustering were performed on the differentially expressed genes identified among the samples which were analyzed using the Dr. Tom platform (BGl, Shenzhen, China) [31].

### 4.11. Statistical Analysis

Statistical analyses were performed using GraphPad Prism 8 software (GraphPad Software, Inc., San Diego, CA, USA). For a two-group comparison, normally distributed datasets underwent unpaired *t* tests. For the comparison of more than two group, one-way ANOVA tests were performed. The significant differences between groups were defined as * *p* < 0.05, ** *p* < 0.01, *** *p* < 0.001, and **** *p* < 0.0001. The error bars represent the standard error of the mean (SEM) of at least three independent experiments.

## 5. Conclusions

In conclusion, our findings underscore the indispensable role of SHMT2 in the normal development of erythroid cells in mice. We provide a comprehensive analysis of the mechanisms that lead to erythroid developmental arrest due to SHMT2 deficiency, elucidating the underlying pathophysiological processes. Additionally, we successfully established an adult murine model of anemia, which offers a novel platform for future investigations into the etiology and treatment of anemia and polycythemia associated with deficiencies in folate and one-carbon metabolism. These insights highlight SHMT2 as a significant therapeutic target for developing interventions aimed at ameliorating these hematological disorders.

## Data Availability

The sequencing data reported in this article have been deposited in the NCBI Sequence Read Archive (SRA) under accession number PRJNA1163286 (accessed on 21 September 2024). The raw data supporting the conclusions of this article will be made available by the authors on request.

## Supplementary Materials

The following supporting information can be downloaded at: https://www.mdpi.com/article/10.3390/ijms252011072/s1.
