# Supplementary material for: Serine Hydroxymethyltransferase 2 Deficiency in the Hematopoietic System Disrupts Erythropoiesis and Induces Anemia in Murine Models
Source: Int J Mol Sci. 2024 Oct 15;25(20):11072. doi: 10.3390/ijms252011072 (PMC11508403; doi:10.3390/ijms252011072)
Supplement: Supplementary file 1 [file ijms-25-11072-s001.zip › ijms-3238942-supplementary.pdf]

Supplementary Table S1. Primers used in this study.		
**Primer set**	**Forward**	**Reverse**
SHMT2-sgRNA	ATGGAGGTATTCATGTCCGGAGG	ATGAACGTTGAGGACAGTTGAGG
SHMT2-5'-loxP	ACACAACTCTTTCCAAGAACTGCTTA	AGGAACAAAATGACCATCCTAAGGT
SHMT2-3'-loxP	CAGCAGCCCTCTGATCAACTTCTCC	TATGCCTGGCTCTTGCCCTAAATGG
Vav1-Cre	AGATGCCAGGACATCAGGAACCTG	ATCAGCCACACCAGACACAGAGATC
SHMT2	TGGCAAGAGATACTACGGAGG	GCAGGTCCAACCCCATGAT
ACTB	TGTGATGGTGGGAATGGGTCAG	TTTGATGTCACGCACGATTTCC

Supplementary Table S2. Antibodies used in this study.				
**Primary Antibody**				
**Antibody**	**Company**	**Product Code**	**Ig Species**	
CD45	Abcam	ab10558	Mouse	
SHMT2	Proteintech	11099-1-AP	Rabbit	
**Conjugated antibody**				
**Antibody**	**Company**	**Product Code**	**Ig Species**	
CD71-PE	BD	553267	Rat	
Ter119-APC-cy7	BD	560509	Rat	
CD117(C-KIT) -APC	BD	553356	Rat	
CD117(C-KIT) -PE-cy7	BD	558163	Rat	
7AAD	BD	559925		
CD3e-FITC	eBioscience	11-0032-82	Rat	
CD45R(B220)-FITC	eBioscience	11-0452-86	Rat	
CD11b-FITC	eBioscience	11-0112-86	Rat	
DRAQ5	Thermo Fisher	62251		
DAPI	Sigma	D9542		
**Secondary Antibody**				
**Antibody**		**Company**	**Product Code**	**Dilution**
Donkey Anti-Mouse IgG H&L (Alexa Fluor® 488)		abcam	ab150105	400
Donkey Anti-Rabbit IgG H&L (Alexa Fluor® 647)		abcam	ab150075	400
